# Large magnetic resonance imaging‐based medial posterior tibial slope is associated with time‐dependent worsening of rotational knee laxity after anterior cruciate ligament reconstruction

**DOI:** 10.1002/jeo2.70552

**Published:** 2025-11-14

**Authors:** Kensaku Abe, Hiroaki Fukushima, Shunta Hanaki, Kyohei Ota, Makoto Kobayashi, Yusuke Kawanishi, Jiro Kato, Satoru Demura, Hideki Murakami, Masahiro Nozaki

**Affiliations:** ^1^ Department of Orthopaedic Surgery Nagoya City University Graduate School of Medical Sciences Nagoya Japan; ^2^ Department of Orthopaedic Surgery, Graduate School of Medical Sciences Kanazawa University Kanazawa Japan; ^3^ Department of Orthopaedic Surgery Nagoya City University Midori Municipal Hospital Nagoya Japan; ^4^ Department of Orthopaedic Surgery Ogaki Municipal Hospital Ogaki Japan; ^5^ Department of Orthopaedic Surgery Kasugai Municipal Hospital Kasugai Japan

**Keywords:** acceleration, anterior tibial translation, external rotational angular velocity, inertial sensor, pivot shift test

## Abstract

**Purpose:**

To examine the relationship between posterior tibial slope (PTS) and knee laxity before, during and after anterior cruciate ligament (ACL) reconstruction, using anterior tibial translation (ATT) measured as an arthrometer‐based side‐to‐side difference and rotational laxity assessed during the pivot shift test via clinical grade and inertial sensor–based measurements of tibial acceleration and external rotational angular velocity (ERAV).

**Methods:**

This retrospective cohort study assessed patients who underwent primary ACL reconstruction with subsequent hardware removal. Medial PTS (MPTS), lateral PTS (LPTS) and slope asymmetry ( | MPTS–LPTS | ) were measured using magnetic resonance imaging. Based on a recent review, binary strata were defined as MPTS ≥ 9.05° versus <9.05° and LPTS ≥ 9.55° versus <9.55°, and comparisons were performed accordingly. Knee laxity was evaluated at three time points: preoperatively, at time‐zero (intraoperatively during temporary graft fixation), and postoperatively (at the time of hardware removal), using ATT, pivot shift grade, acceleration and ERAV. Variables with *p* < 0.1 in univariate analysis, along with key PTS factors, were entered into multivariate linear or ordinal logistic regression models. Cohen's *d* was calculated for binary predictors of continuous outcomes, with |*d* | ≥0.5 indicating a moderate or greater effect size; odds ratios (ORs) with 95% confidence intervals were reported.

**Results:**

A total of 106 patients (24.3 ± 10.3 years; 37.7% male; 22.4 ± 2.7 kg/m^2^) were analysed. Large MPTS (≥9.05°) was significantly associated with increased acceleration (*p* = 0.017, |*d* | = 0.62) and ERAV (*p* = 0.004, |*d* | = 0.59) at the postoperative time point (hardware removal, 1.6 ± 0.6 years after ACL reconstruction). No significant associations with PTS were observed for ATT or pivot shift grade.

**Conclusion:**

Large MPTS was associated with increased postoperative rotational laxity, although no laxity was noted intraoperatively during temporary fixation. These results indicate that certain tibial slope morphologies may drive the onset of laxity. Preoperative assessment may help identify at‐risk patients and optimize surgical strategy.

**Level of Evidence:**

Level IV.

**Abbreviations**
ACL: anterior cruciate ligament
ALL: anterolateral ligament
ATT: anterior tibial translation
BMI: body mass index
CI: confidence interval
IKDC: International Knee Documentation Committee
LET: lateral extra‐articular tenodesis
LPTS: lateral posterior tibial slope
MPTS: medial posterior tibial slope
MRI: magnetic resonance imaging
OR: odds ratio
PTS: posterior tibial slope
SSD: side‐to‐side difference
SSR: side‐to‐side ratio

## INTRODUCTION

Residual instability after anterior cruciate ligament (ACL) reconstruction, identified by a positive pivot shift test, occurs in 8%–33% of cases [3, 14, 22], and failure rate ranges from 5.2% to 25.5% [13, 24, 33]. Contributing factors include femoral tunnel positioning [29], graft selection [30], patient age [40], sex [27], secondary stabilizers [18], omission of anterolateral ligament (ALL) reconstruction [38] and bone morphology [2]. Among these, posterior tibial slope (PTS) has recently gained increasing attention as a potential risk factor for both primary and recurrent ACL injuries [21]. In this study, anterior tibial translation (ATT) denotes an arthrometer‐based side‐to‐side difference (SSD). Across prior literature, ‘anterior laxity/ATT’ has been measured with heterogeneous methods. ATT rises significantly when the PTS exceeds 12° [8]. Moreover, several studies have indicated that PTS affects not only the ATT [6, 7, 28] but also rotational laxity, which may be influenced by medial PTS (MPTS) [42] and lateral PTS (LPTS) [17, 34]. In addition, MPTS and LPTS asymmetry have recently been implicated as a contributor to rotational laxity [17].

Traditionally, rotational instability has been assessed subjectively via the manual pivot shift test [1, 23, 26, 32]. Recent advances have facilitated its quantitative assessment through various technologies, including inertial sensors with validated accuracy [20, 31, 42].

While several studies have investigated preoperative and postoperative laxity, few have assessed intraoperative knee stability at the time‐zero phase, with limited exceptions such as the reports by Kawanishi et al. [19] and Sheean et al. [35]. Evaluating laxity at time‐zero (i.e., during intraoperatively temporary graft fixation) allows for identification of temporal changes in stability. This approach may distinguish newly developed postoperative laxity (measured at the time of hardware removal) from residual preoperative instability.

The clinical relevance of identifying slope‐related anatomic risk factors has been emphasized to aid preoperative risk stratification and interpretation of postoperative laxity, and this study was undertaken to address the gap that only a few investigations have quantified rotatory laxity across preoperative, intraoperative time‐zero and postoperative time points within the same cohort.

The aim of this study was to assess the influence of MPTS and LPTS on knee laxity using inertial sensors across three time points—preoperatively, time‐zero and postoperatively. It was hypothesized that a steeper PTS and greater slope asymmetry would be associated with increased postoperative rotational laxity, whereas no association would be observed at time‐zero. Compartment‐specific effects (medial vs. lateral slopes) were designated as exploratory. Time‐dependent changes in rotatory laxity were hypothesized on the basis of clinical observations that, despite satisfactory stability at the end of surgery after final graft fixation, some knees were subsequently found to exhibit rotational laxity at follow‐up.

## MATERIALS AND METHODS

This study was conducted in accordance with the Declaration of Helsinki and received approval from the Institutional Review Board (IRB) of the Ethics Committee of Nagoya City University (IRB number: C‐T2024‐0375). Written informed consent was obtained from all participants.

### Patients selection

Patients who underwent primary ACL reconstruction between June 2016 and November 2024 were retrospectively reviewed. The inclusion criteria consisted of patients who subsequently underwent hardware removal following ACL reconstruction. At our institution, tibial staple removal is routinely offered approximately 1 year postoperatively to patients experiencing discomfort due to palpable hardware or based on personal preference. This procedure was performed independently of research objectives. Patients were excluded if they had associated ligament injuries, underwent concurrent osteotomy, had incomplete data, underwent ALL reconstruction (in this cohort, all lateral extra‐articular procedure cases involved ALL reconstruction), experienced contralateral ACL injury, experienced graft re‐rupture prior to hardware removal or had a Tegner activity scale score of 5 or lower. ALL reconstruction was excluded because it has been shown to enhance postoperative knee stability and may mask intrinsic laxity [38]. Patients with lower activity levels were excluded to avoid underestimation of true postoperative laxity, whereas those with a Tegner activity scale of 6 or higher were included due to the increased risk of instability associated with high activity levels [39, 43]. All ACL reconstructions in the final cohort were performed using the double‐bundle technique with hamstring tendon autografts.

### Laxity evaluation

**Figure 1:**
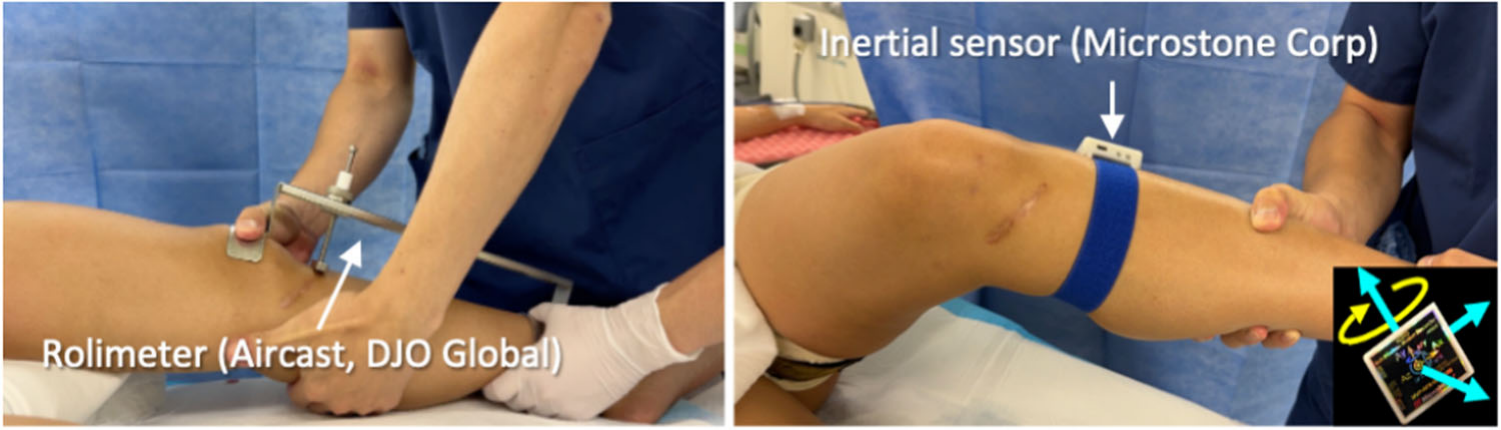
In the left panel, anterior tibial translation (ATT) is measured using a Rolimeter. In the right panel, a pivot shift test is performed, and an inertial sensor is attached to measure the tibial acceleration and external rotational angular velocity.

In this study, knee laxity under general anaesthesia was assessed according to previous reports [11, 19, 20], using ATT and pivot shift test, as objective variables. ATT was evaluated by the SSD using a Rolimeter arthrometer (Aircast, DJO Global) at the point of maximum tension by manual anterior pull in the mildly flexed knee position, and the pivot shift test was assessed subjectively evaluated using the International Knee Documentation Committee (IKDC) grade and objectively evaluated using an inertial sensor (MVP‐RF8‐BC; MicroStone) to measure tibial acceleration (m/s^2^) and external rotational angular velocity (ERAV; °/s) [19, 20, 31], and reported as the side‐to‐side ratio (SSR) (Figure 1). All pivot‐shift manoeuvres were performed by a single senior orthopaedic surgeon (M. N.), using the same inertial‐sensor model and acquisition protocol as in our prior work [11, 19, 20]. Although inter‐ and intrarater reliability were not reassessed within the present cohort, high intraobserver intraclass correlation coefficients have been reported for quantitative laxity measurements during the pivot‐shift test using an inertial sensor [31]. The measurements were taken at three time points—preoperatively, time‐zero (intraoperatively during temporary graft fixation), and postoperatively (at the time of hardware removal). At time zero, grafts were tensioned and temporarily fixed prior to final fixation, following the standard intraoperative protocol used at our institution. This technique, including the timing and fixation procedure, has been described in detail previously [11]. Since all cases demonstrated grade 0 pivot shift at time zero, evaluation at that time was omitted.

### Magnetic resonance imaging (MRI) evaluation of tibial slope

MPTS and LPTS were measured via MRI according to a previously published protocol [12]. On sagittal MRI, the tibial diaphyseal axis was identified, and a line perpendicular to this axis was constructed at the anterior peak of each plateau. The slope was then measured as the angle between this perpendicular and a line tangential to the subchondral surface of the respective plateau.

### Variables and operational definitions

The explanatory variables were age, sex, body mass index (BMI; kg/m^2^), presence or absence of medial meniscus repair, presence or absence of lateral meniscus repair, time from injury to ACL reconstruction (months), time from ACL reconstruction to hardware removal (years), MPTS (°), LPTS (°) and slope asymmetry which was defined as the absolute difference between MPTS and LPTS (| MPTS − LPTS |) (°), regardless of direction, to reflect the extent of posterior slope imbalance. All explanatory variables were binarized for statistical analysis. Age (< 20 years = 0, ≥20 years = 1), sex (female = 0, male = 1), BMI (<22.3 kg/m^2^ = 0, ≥22.3 kg/m^2^ = 1), lateral meniscus repair (no = 0, yes = 1), medial meniscus repair including ramp lesion (no = 0, yes = 1), time from injury to ACL reconstruction (<2.9 months = 0, ≥2.9 months = 1) and time from ACL reconstruction hardware removal (<1.5 years = 0, ≥1.5 years = 1) were dichotomized using the respective median values in the current cohort. In contrast, PTS variables were binarized based on previous literature: MPTS (<9.05° = 0, ≥9.05° = 1) and LPTS (<9.55° = 0, ≥ 9.55° = 1) [5]. Slope asymmetry |MPTS − LPTS| was also binarized using the cohort median value (|MPTS − LPTS | <1.6° = 0, ≥1.6° = 1).

### Statistical analysis

Statistical analyses were conducted using EZR (Saitama Medical Center, Jichi Medical University, Saitama, Japan), a graphical user interface for R (The R Foundation for Statistical Computing). This platform is a modified version of R Commander, designed to add statistical functions frequently used in biostatistics [16].

Univariate comparisons were performed using the Mann–Whitney *U* test to assess associations between binary explanatory variables and continuous outcomes, including ATT, acceleration and ERAV. The pivot shift grade, treated as an ordinal outcome, was initially analysed using the Mann–Whitney *U* test. Explanatory variables with *p* < 0.1 in univariate analysis, along with PTS‐related variables (MPTS, LPTS or |MPTS − LPTS | ), were included in multivariate models. Multivariate linear regression was used for continuous outcomes (ATT, acceleration and ERAV), while multivariate ordinal logistic regression was applied for pivot shift grade. To improve interpretability in this retrospective study, effect sizes were calculated using Cohen's *d* [4] for binary explanatory variables in relation to continuous outcomes, as an a priori power analysis was not feasible. The sample size reflected all eligible cases during the study period, and no post‐hoc power analysis was performed. Cohen's *d* was calculated only for variables with *p* < 0.1 in the multivariate linear regression analysis. Following conventional guidance, absolute values of *d*, that is, 0.2, 0.5 and 0.8 were interpreted as small, moderate and large, respectively; in this study, |*d* | ≥0.5 was prespecified as clinically meaningful (‘effect present’). Absolute mean differences with 95% confidence intervals (CIs) were reported alongside *d* to aid clinical interpretation. These were reported regardless of statistical significance for the key morphological factors (MPTS, LPTS and |MPTS − LPTS | ). For ordinal outcomes, odds ratios (ORs) and 95% confidence intervals (CIs) were reported.

To assess measurement reliability, MPTS and LPTS were measured by two independent raters using identical MRI images in 30 randomly selected cases (>20% of the cohort). Rater 2 repeated the measurements after an interval exceeding 3 months. Intraclass correlation coefficients (2,1) demonstrated good to excellent reliability: 0.782 (95% CI: 0.570–0.914) and 0.875 (95% CI: 0.736–0.955) for inter‐rater (MPTS and LPTS) and 0.957 (95% CI: 0.908–0.980) and 0.987 (95% CI: 0.972–0.993) for intra‐rater, respectively.

## RESULTS

**Figure 2:**
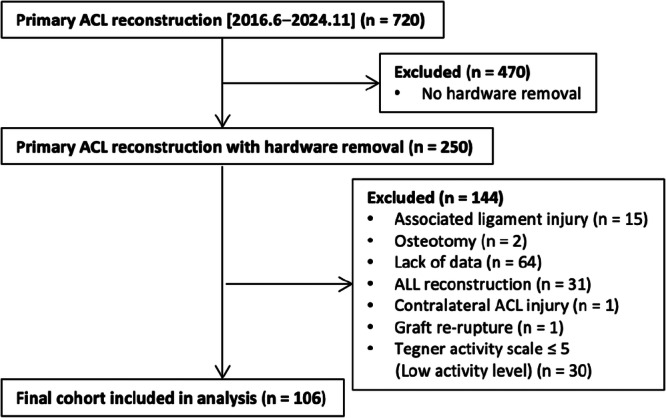
Flow diagram of patient selection. Numbers at each step indicate exclusions by reason; the final cohort comprised 106 patients. ACL, anterior cruciate ligament; ALL, anterolateral ligament.

A total of 720 patients underwent primary ACL reconstruction during the study period. Of these, 470 were excluded due to no hardware removal, and 144 were further excluded based on the predefined criteria. Ultimately, 106 patients were included in the final analysis (Figure 2). The demographic and morphological characteristics of the included patients are summarized in Table 1.

**Table 1:** Demographic and morphological characteristics.

Variable	Value (Mean ± SD or *n* [%])	Range (continuous variables)
Age (years)	24.3 ± 10.3	12 − 54
Sex (male)	40 (37.7)	
BMI (kg/m^2^)	22.4 ± 2.7	17.6 − 30.0
MM repair	48 (45.3)	
LM repair	37 (34.9)	
Time from injury to ACLR (months)	8.4 ± 19.8	1.0 − 144.0
Time to hardware removal (years)	1.6 ± 0.6	0.9 − 4.1
MPTS (°)	5.9 ± 2.9	0.0 − 15.0
LPTS (°)	5.9 ± 2.7	−0.5 − 12.2
|MPTS ‐ LPTS| (°)	2.1 ± 1.7	0.0 − 9.0

Abbreviations: ACLR, anterior cruciate ligament reconstruction; BMI, body mass index; LM, lateral meniscus; LPTS, lateral posterior tibial slope; MM, medial meniscus; MPTS, medial posterior tibial slope; SD, standard deviation.

### Univariate and multivariate analyses

In the univariate analysis, a significant association was found only between LPTS and increased acceleration at time‐zero (*p* = 0.031); no other significant relationships were identified for PTS‐related parameters across the time points.

**Figure 3:**
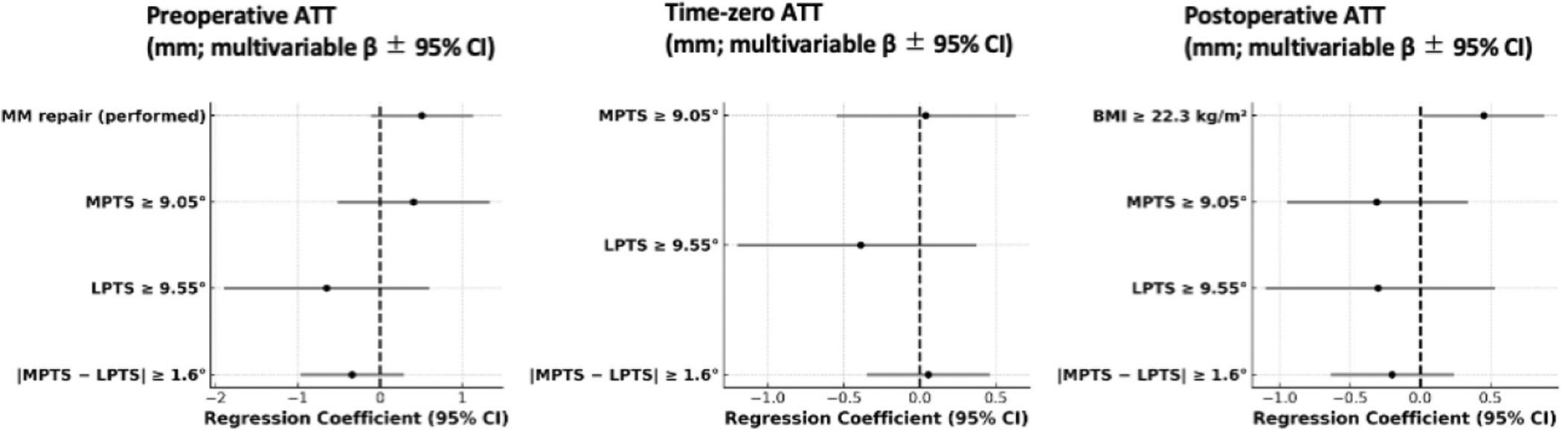
Multivariable linear regression for anterior tibial translation (ATT, mm). Forest plots display regression coefficients (β) with 95% confidence intervals (vertical line = β = 0). ATT was measured as an arthrometer‐based side‐to‐side difference. Panels: left = preoperative, centre = intraoperative (time‐zero), right = postoperative (hardware removal). Morphological predictors were prespecified as MPTS ≥ 9.05°, LPTS ≥ 9.55° and |MPTS − LPTS | ≥ 1.6°. Binary variables were coded 1 = present, 0 = absent. *n* = 106. ATT, anterior tibial translation; CI, confidence interval; LPTS, lateral posterior tibial slope; MPTS, medial posterior tibial slope.

**Figure 4:**
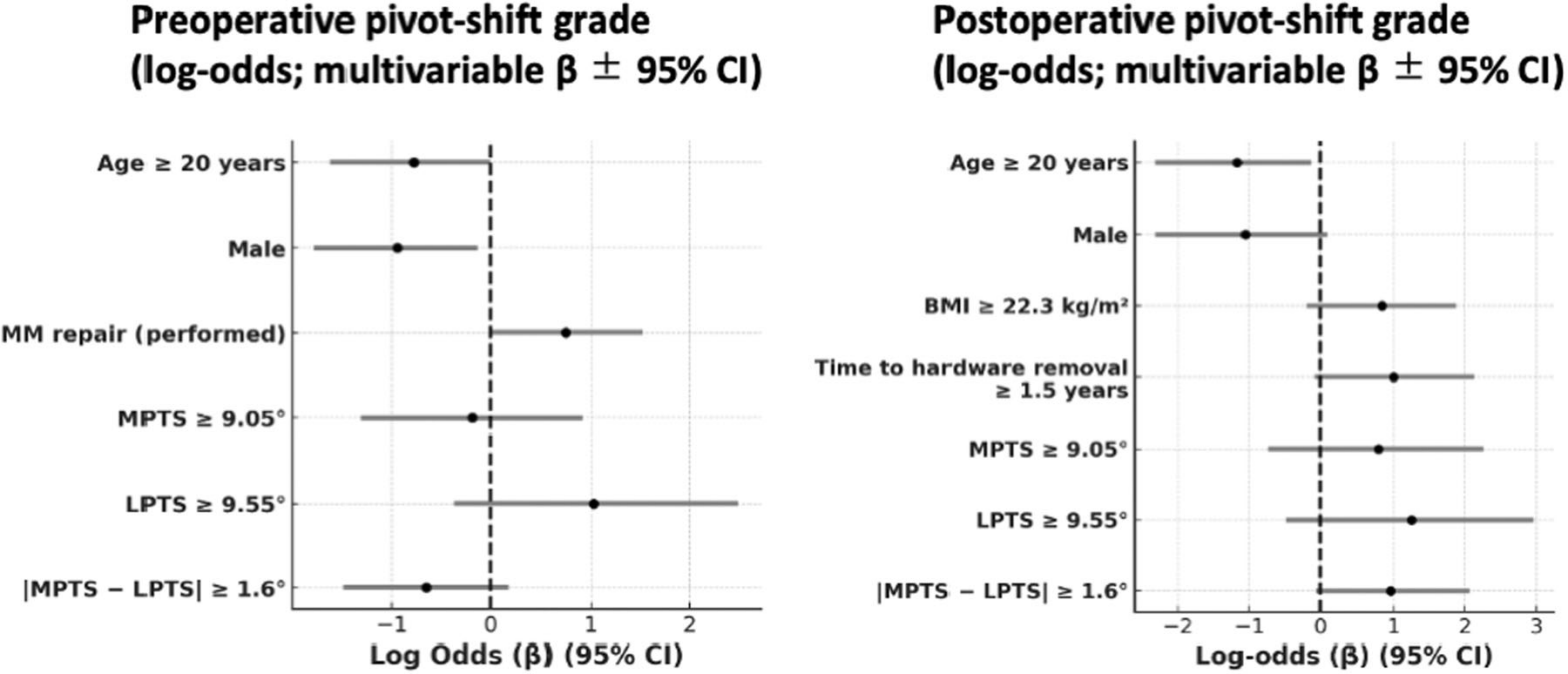
Ordinal logistic regression for pivot‐shift grade (IKDC). Forest plots display log‐odds coefficients (*β* = ln[OR]) with 95% confidence intervals (vertical line = *β* = 0). Panels: left = preoperative, right = postoperative (hardware removal). Pivot‐shift grade was assessed clinically (IKDC). Morphological predictors were prespecified as MPTS ≥ 9.05°, LPTS ≥ 9.55° and |MPTS − LPTS | ≥ 1.6°. Binary variables were coded 1 = present, 0 = absent. *n* = 106. CI, confidence interval; IKDC, International Knee Documentation Committee; LPTS, lateral posterior tibial slope; MPTS, medial posterior tibial slope.

**Figure 5:**
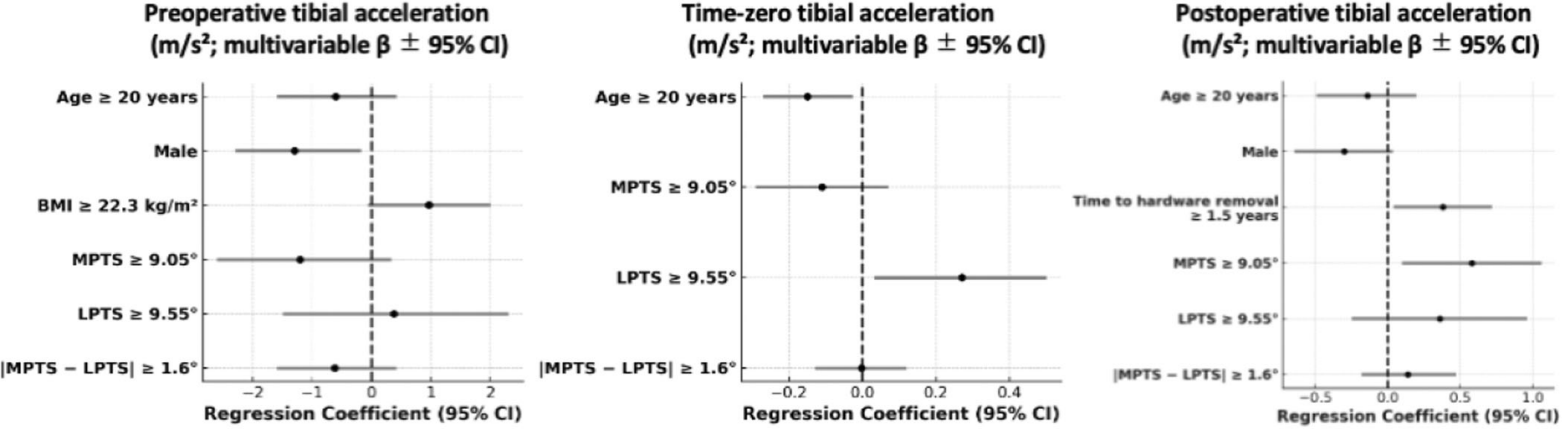
Multivariable linear regression for pivot‐shift tibial acceleration (m/s²). Forest plots display regression coefficients (*β*) with 95% confidence intervals (vertical line = *β* = 0). Tibial acceleration was measured with an inertial sensor during the pivot‐shift test. Panels: left = preoperative, centre = intraoperative (time‐zero), right = postoperative (hardware removal). Morphological predictors were prespecified as MPTS ≥ 9.05°, LPTS ≥ 9.55° and |MPTS − LPTS | ≥ 1.6°. Binary variables were coded 1 = present, 0 = absent. *n *= 106. CI, confidence interval; LPTS, lateral posterior tibial slope; MPTS, medial posterior tibial slope.

**Figure 6:**
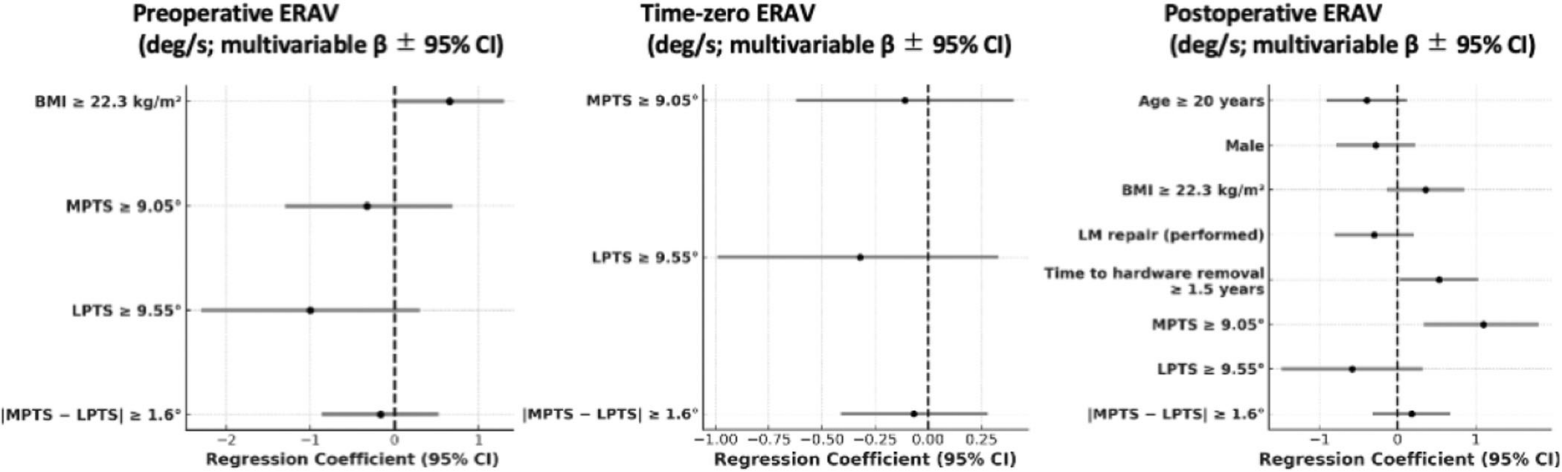
Multivariable linear regression for external rotational angular velocity (ERAV, deg/s). Forest plots display regression coefficients (*β*) with 95% confidence intervals (vertical line = *β* = 0). ERAV was measured with an inertial sensor during the pivot‐shift test. Panels: left = preoperative, centre = intraoperative (time‐zero), right = postoperative (hardware removal). Morphological predictors were prespecified as MPTS ≥ 9.05°, LPTS ≥ 9.55° and |MPTS − LPTS | ≥ 1.6°. Binary variables were coded 1 = present, 0 = absent. *n* = 106. CI, confidence interval; ERAV, external rotational angular velocity; LPTS, lateral posterior tibial slope; MPTS, medial posterior tibial slope.

Results from the multivariate analysis with statistically significant predictors (*p* < 0.05) and/or moderate effect sizes ( | Cohen's *d* | ≥0.5) are summarized below. Detailed results are provided in Tables S1–S4 and Figures 3, 4, 5, 6. Exploratory correlations among ATT, pivot‐shift grade, acceleration and ERAV were weak to moderate overall, with ATT showing only small correlations with rotational indices, whereas pivot‐shift grade correlated moderately with sensor‐based metrics (Table S5).

### ATT

In the multivariate analysis, no PTS‐related parameters (MPTS, LPTS or |MPTS − LPTS | ) showed significant associations with ATT at any of the three time points. None of the remaining variables showed a statistically significant effect with a moderate or larger effect size.

### Pivot shift grade

The pivot shift grade was analysed using ordinal logistic regression. No PTS‐related variables showed statistically significant associations with pivot shift grade. Preoperatively, female sex was significantly associated with higher pivot shift grades (odds ratio [OR] = 0.39, 95% confidence interval [CI]: [0.17–0.88], *p* = 0.026). Postoperatively, younger age (<20 years) was significantly associated with increased pivot shift grade (OR = 0.31, 95% CI: [0.099–0.88], *p* = 0.033).

### Acceleration

Preoperatively, no PTS‐related variables showed significant associations with acceleration. At time‐zero, greater acceleration was observed in participants with LPTS ≥ 9.55° (*p* = 0.027, 95% CI: [0.032–0.50], |Cohen's *d* | = 0.72), indicating a potential contribution of increased lateral PTS to early postoperative dynamic laxity. Postoperatively, MPTS ≥ 9.05° was significantly associated with increased acceleration (*p* = 0.017, 95% CI: [0.10–1.06], |Cohen's *d* | = 0.62), suggesting that a steeper medial slope may contribute to residual dynamic laxity following ACL reconstruction. Among the other variables, female sex showed significant associations with increased preoperative acceleration (*p* = 0.023, 95% CI: [−2.33–−0.18], |Cohen's *d* | = 0.57).

### ERAV

Preoperatively and at time zero, no PTS‐related variables showed significant associations with ERAV. Postoperatively, MPTS ≥ 9.05° was significantly associated with increased ERAV (*p* = 0.0041, 95% CI: [0.34–1.77], |Cohen's *d* | = 0.59), suggesting that a steeper MPTS may contribute to residual rotational laxity following ACL reconstruction. No other variables showed both statistical significance and a moderate or greater effect size.

## DISCUSSION

The most notable finding of this study was that a steeper MPTS was significantly associated with increased rotational laxity at the time of hardware removal, despite no such association being evident at time‐zero. This dynamic change was objectively detected using inertial sensors, highlighting the potential role of MPTS in the development of delayed rotational laxity.

Beyond the tibial slope, associations were also observed for patient and temporal factors. In selected models, younger age, female sex and a longer interval from ACL reconstruction to hardware removal were associated with greater measures of laxity (consistent with sex‐ and maturation‐related differences reported in prior work [36]). These findings should be interpreted cautiously, given the retrospective design and potential residual confounding; nevertheless, they are clinically plausible in light of activity‐related loading and postoperative tissue adaptation, with effect sizes generally in the small‐to‐moderate range (Tables S1–S4).

All reconstructions in this cohort were performed using a standardized double‐bundle technique, which may better constrain anterior and rotational laxity than single‐bundle constructs; consequently, associations between tibial slope and laxity may have been attenuated. A direct comparison with single‐bundle ACLR was not performed; therefore, this interpretation remains speculative.

As shown in previous reports demonstrating that PTS > 12° leads to increased preoperative ATT [8], several studies have reported a strong relationship between increased PTS and pre‐ or postoperative ATT [6, 7, 10, 25]. Although damage to secondary stabilizers, such as the meniscus, has been reported not to correlate with increased ATT [18], suggesting that the influence of other non‐PTS factors may be limited, our study found no significant association between PTS and ATT at any time point, which is a major difference from prior findings. In this study, cutoff values of 9.05° for MPTS and 9.55° for LPTS were adopted based on a recent review article [5]. In contrast, Li et al. identified lower thresholds, MPTS > 5.6° and LPTS > 3.8° as risk factors for increased ATT [25]. In addition, ATT was quantified as an arthrometer‐based SSD under general anaesthesia, whereas prior studies often used stress radiographs or navigation‐based, weight‐bearing measures, which are not interchangeable. A standardized double‐bundle technique and frequent meniscal repairs in this cohort may also have reduced anterior translation, attenuating slope–ATT associations at follow‐up. Finally, a relatively narrow range of ATT and the use of dichotomized PTS (with potential nonlinearity) may have limited detection of small effects after multivariable adjustment.

Recently, the influence of PTS on rotational laxity assessed via the pivot shift test has gained attention. While various mechanisms have been proposed, some studies—consistent with our results—have reported that increased MPTS [42] and asymmetry between MPTS and LPTS contribute to rotational laxity [17]. Others have emphasized the role of LPTS [17, 34]. All of these reports assessed preoperative rotational laxity. Although no association was observed preoperatively in our cohort, rotational laxity was evaluated at three time points, and it was found that a steeper MPTS was associated with greater postoperative rotational laxity. By contrast, slope asymmetry (| MPTS − LPTS |) did not show a statistically significant association in our analyses. Conversely, increased LPTS was associated with greater acceleration at time‐zero, suggesting a potential contribution to early postoperative dynamic laxity in this study. However, no association was observed postoperatively, indicating that LPTS may not influence long‐term residual laxity. Still, due to its short‐term impact, LPTS should be closely monitored in clinical evaluations.

The particular relevance of MPTS to rotational laxity may be explained by compartmental mechanics. The medial tibial plateau's concavity, combined with a steeper medial posterior slope, can increase anterior shear in the medial compartment under axial load and thereby generate a coupled internal rotation of the tibia relative to the femur. Immediately after reconstruction, a double‐bundle construct may constrain anterolateral translation, which could account for the limited time‐zero associations; however, with postoperative tissue adaptation and graft viscoelastic changes, the medial slope effect may become more evident at follow‐up. By contrast, LPTS exhibited an acute association at time‐zero only in this cohort and no postoperative association, suggesting that its contribution to long‐term residual rotational laxity is uncertain in the present setting.

Clinically, an increased PTS has been reported to be associated with both primary ACL injury and graft re‐rupture risk [5, 15]. Particularly regarding re‐injury, postoperative increases in ATT and rotational laxity, as observed in previous reports and supported by our findings, are believed to contribute to failure [6, 25]. Although lateral extra‐articular tenodesis (LET) and ALL reconstruction have been reported to improve rotational stability and reduce failure rates after ACL reconstruction [9, 37, 41], these adjunctive procedures were not evaluated in our study; their roles in the context of steep PTS warrant dedicated investigation rather than inference from the present data.

This study has certain limitations. First, its retrospective design may introduce selection bias and limit causal inference. Because hardware removal was performed in a subset (250/720) and the analytic cohort was further restricted (*n* = 106), generalizability to all ACL reconstructions is uncertain; however, the primary exposures (preoperative MRI–based slopes/asymmetry) are anatomic, and time‐zero measurements were intraoperative, making broader applicability biologically plausible. Second, while this study focused on PTS as a morphological factor, other bony features [2] including intercondylar notch width, posterior condylar offset, or lateral tibial plateau size and femoral and tibial tunnel positions were not evaluated and could influence postoperative rotational stability. Third, although the cutoff values for MPTS and LPTS were based on a recent review article [5], these thresholds vary across studies, which may have influenced the results. Fourth, the ‘postoperative’ assessment occurred at hardware removal, and the interval varied across patients. Fifth, inter‐ and intrarater reliability of the inertial‐sensor pivot‐shift measures were not reassessed to minimize procedure time under general anaesthesia; variability was mitigated by a single‐operator protocol using a previously validated device and procedure. In addition, no patient‐reported outcome measures (PROMs) or revision events were collected; therefore, it remains unclear whether the observed differences in rotational laxity translate into differences in clinical outcomes or reoperation rates. Finally, although effect sizes were used to supplement statistical power, the relatively small sample size may have limited the detection of subtle associations.

## CONCLUSION

Increased MPTS was significantly associated with greater postoperative rotational laxity following ACL reconstruction, as reflected by higher inertial‐sensor acceleration and ERAV during the pivot‐shift test. These findings highlight the potential relevance of tibial slope anatomy—particularly increased MPTS—when interpreting postoperative laxity. Confirmation in broader ACL reconstruction populations is warranted.

## AUTHOR CONTRIBUTIONS

All authors contributed to the conception and design of the study. The material preparation, data collection, and analyses were performed by Kensaku Abe, Hiroaki Fukushima, Shunta Hanaki, Kyohei Ota, Yusuke Kawanishi and Jiro Kato. The first draft of the manuscript was written by Kensaku Abe, and all authors commented on the previous versions of the manuscript. All authors have read and approved the final version of the manuscript.

## CONFLICT OF INTEREST STATEMENT

The authors declare no conflict of interest.

## ETHICS STATEMENT

This study was approved by the Institutional Review Board (IRB) of the Ethics Committee of Nagoya City University (IRB number: C‐T2024‐0375). In this study, all patients or their families were informed about the “comprehensive study on the effectiveness of treatment for anterior cruciate ligament injury” at the time of treatment and asked whether they wished to participate in the study or not, and the data of patients who gave their consent were used retrospectively.

## Supporting information

Supplemental Table S1.

Supplemental Table S2.

Supplemental Table S3.

Supplemental Table S4.

Supplemental Table S5.

## Data Availability

The datasets generated and/or analysed during the current study are not publicly available due to the inclusion of personal health information and institutional restrictions but are available from the corresponding author on reasonable request.
